# Tumour Necrosis Factor‐α Promotes Pyroptosis in Diabetic Liver Injury via the HMGB1/TLR4/MyD88/NF‐κB Pathway

**DOI:** 10.1111/jcmm.71220

**Published:** 2026-05-31

**Authors:** Dayin Chen, Jiajun Cheng, Bin Ni, Ruixin Zhu, Wei Wu, Minghui Zhang, Yihe Cao, Zhimei Jiang, Hongyu Sui

**Affiliations:** ^1^ Department of Urology First Affiliated Hospital, Jiamusi University Jiamusi China; ^2^ Department of Physiology, Basic Medical College Jiamusi University Jiamusi Heilongjiang China; ^3^ Rehabilitation Medicine College Jiamusi University Jiamusi China

**Keywords:** diabetic liver injury, HMGB1, NLRP3 inflammasome, pyroptosis, streptozotocin, TLR4 signallingsignaling, TNF‐α

## Abstract

Diabetic liver injury (DLI) is a serious inflammatory complication, with tumour necrosis factor‐alpha (TNF‐α) recognized as a key pro‐inflammatory cytokine. This study aimed to investigate the protective effect of TNF‐α inhibition on DLI and to elucidate the underlying molecular mechanisms, focusing on the novel role of pyroptosis. An integrated experimental approach was employed, commencing with in vitro studies in HepG2 hepatocytes to dissect the signalling pathway, followed by in vivo validation in Sprague–Dawley rats. In vitro assessments included the expression of pyroptosis‐related proteins (caspase1, Gasdermin D [GSDMD]), high mobility group protein B1 (HMGB1), and components of the Toll‐like receptor 4 (TLR4)/myeloid differentiation factor 88 (MyD88)/nuclear factor‐κB (NF‐κB) pathway under high glucose conditions with or without TNF‐α inhibition. In vivo measurements comprised liver function tests alanine aminotransferase (ALT), aspartate aminotransferase (AST), alkaline phosphatase (ALP), triglycerides (TG), liver histopathology, and analysis of the HMGB1/TLR4/MyD88/NF‐κB pyroptosis axis in liver tissues. In vitro, TNF‐α inhibition mitigated high glucose‐induced hepatocyte pyroptosis and suppressed HMGB1 release and TLR4/MyD88/NF‐κB signalling; pharmacological blockade of HMGB1 replicated these effects. In vivo, recombinant human TNF receptor‐II:Fc fusion protein (rhTNFR:Fc) significantly improved liver function and histopathology in diabetic rats without affecting blood glucose levels, while also downregulating the expression of HMGB1/TLR4/MyD88/NF‐κB pathway components and key pyroptosis markers such as NOD‐like receptor family pyrin domain containing 3 (NLRP3). In conclusion, TNF‐α promotes DLI by driving hepatocyte pyroptosis through the HMGB1/TLR4/MyD88/NF‐κB signalling axis. Inhibition of TNF‐α confers potent hepatic protection independent of glycemic control, identifying it as a promising therapeutic strategy for DLI.

## 1 Introduction

Diabetes Mellitus (DM) has emerged as a significant healthcare concern due to its potential to induce various complications, including liver disease, retinopathy, and renal failure. As a common endocrine and metabolic disorder, DM is characterized by chronic inflammation. Over recent decades, the global prevalence of diabetes has been continuously increasing. According to data released by the International Diabetes Federation (IDF), the number of individuals with diabetes worldwide reached 537 million in 2021 and is projected to rise to 780 million by 2045 [1]. Diabetic Liver Disease refers to histopathological and functional alterations in the liver induced by diabetes and represents a chronic complication of DM. Under persistent hyperglycemic conditions, disturbances in glucose and lipid metabolism can lead to excessive accumulation of glycogen and lipids, resulting in hepatocellular injury. Type 1 Diabetes Mellitus (T1DM) is a key risk factor for diabetic liver disease, attributable to the liver's central role in regulating glucose and lipid metabolism, as it stores or releases glucose in response to metabolic demands [2]. Although DLD has been extensively studied, the majority of research has focused on Type 2 Diabetes Mellitus (T2DM) [3]. Therefore, it is of considerable importance to conduct in‐depth investigations into liver injury in patients with T1DM. Studies have shown that hyperglycemia can trigger inflammatory responses in hepatocytes, leading to severe cellular damage through mitochondrial oxidative stress, endoplasmic reticulum stress, and impaired lysosomal autophagy, ultimately contributing to liver dysfunction [4, 5, 6]. Currently, inflammatory responses are recognized as playing a central role in the pathophysiology of DLD, serving not only as a marker of subsequent injury but also as a key driver in the initiation and progression of the disease [7].

Pyroptosis is one of the primary pathways that induce and exacerbate inflammation. In 2001, Cookson et al. first described a form of cell death dependent on caspase1 in macrophages and termed it “pyroptosis” [8]. During pyroptosis, the nucleotide‐binding oligomerization domain‐like receptor protein 3 (NLRP3) combines with pro‐caspase1 to form an inflammasome. The inflammasome processes caspase1 into cleaved caspase1 (c‐caspase1) [9]. Furthermore, the cleavage and activation of the pore‐forming effector protein GSDMD by activated caspase is a critical step in inducing pyroptosis, during which pro‐inflammatory cytokines such as Interleukin‐1*β* (IL‐1*β*) and Interleukin‐18 (IL‐18) are released from damaged cells. Immune cells are activated by these pro‐inflammatory cytokines, which in turn can induce pyroptosis, establishing a vicious cycle [10]. Recent scientific research has revealed that pyroptosis occurs extensively in patients with liver diseases [11, 12]. In the context of liver injury, pyroptosis is often associated with severe inflammatory responses and infection. Studies indicate that excessive pyroptosis accelerates the progression of DLD, leading to sustained damage to intrinsic parenchymal cells in the liver [13]. In light of this, inhibiting hepatocyte pyroptosis in the early stages of the disease has emerged as an effective strategy to limit the progression of DLD.

TNF‐α, a key biomarker in the early stages of DLD, sensitively reflects the extent of liver damage [14] and is closely associated with pyroptosis [15]. Previous studies have shown that TNF‐α is commonly used as an inducer of pyroptosis in ex vivo experiments, while inhibition of TNF‐α can alleviate pyroptosis in conditions such as acute liver failure, kidney damage, and rheumatoid arthritis [16]. However, the pathological mechanism linking TNF‐α and pyroptosis in DLD remains unclear. Numerous studies indicate that High Mobility Group Box 1 (HMGB1) is not only a core regulatory protein in the inflammatory cascade of Acute Liver Failure (ALF) [17] but also plays a critical role in the pathophysiology of various acute and chronic liver diseases [18]. Extensive research has demonstrated that the TLR4/MyD88/NF‐κB pathway is a major route promoting inflammation and disease progression in liver disorders. Although TNF‐α, HMGB1, and TLR4 signalling are each independently associated with liver pathology, it remains unknown whether they converge into a coordinated pathway driving diabetic liver injury. In summary, this study proposes the following hypothesis: TNF‐α acts as a molecular hub that specifically induces hepatocyte pyroptosis via the HMGB1‐mediated TLR4/MyD88/NF‐κB signalling pathway, thereby exacerbating hepatic inflammation and injury. To validate this mechanism, we applied the specific antagonist rhTNFR:Fc, aiming to elucidate the role and specific mechanisms by which TNF‐α regulates hepatocyte pyroptosis, with the goal of untangling key pathological processes of the disease and identifying potential therapeutic targets.

## 2 Materials and Methods

### 2.1 In Vitro Study of TNF‐α Inducing Pyroptosis in Liver Cells

#### 2.1.1 Cell Culture and Treatment

The human hepatocellular carcinoma cell line HepG2 (SCSP‐510, Cell Bank of Chinese Academy of Sciences) was maintained in high‐glucose DMEM (HyClone) containing 25 mM glucose, supplemented with 10% FBS (Gibco) and 1% penicillin–streptomycin at 37°C with 5% CO_2_. All cells were confirmed mycoplasma‐free. For experiments, HepG2 cells were seeded at 1.5 × 10^5^ cells/mL in 96‐well plates. After 12 h of attachment, cells were treated with additional D‐glucose (Sigma‐Aldrich) at indicated concentrations. Specifically, the basal culture medium already contained 25 mM glucose; to achieve final glucose concentrations of 50, 75, 100, 150, and 200 mM, the medium was supplemented with an additional 25, 50, 75, 125, and 175 mM glucose, respectively. Cells were incubated for 24–72 h under the same culture conditions. To assess protective effects, cells were pretreated for 48 h with rhTNFR:Fc and/or the HMGB1 inhibitor glycyrrhetinic acid (GL, SM6092, Beyotime), followed by 48 h co‐culture with high glucose (75 mM). In all cell‐based experiments, including the CCK‐8 assay, solvent control groups with equal volumes were established. rhTNFR:Fc was dissolved in sterile water for injection to prepare a high‐concentration stock solution, which was subsequently diluted to the working concentration using complete culture medium. The HMGB1 inhibitor was dissolved in DMSO, with a final concentration of ≤ 0.1%. A corresponding solvent control group containing an equal volume of 0.1% DMSO was also established. No significant cytotoxicity was observed in any of these solvent control groups compared to the blank control group. Experimental groups included: Normal control, High glucose, Normal control + HMGB1 inhibitor, High glucose + rhTNFR:Fc, High glucose + HMGB1 inhibitor.

#### 2.1.2 CCK‐8 Viability Assay

Cell viability was assessed using the Cell Counting Kit‐8 (CCK‐8, MA0218; Meiluncell, China). For the CCK‐8 assay, cells were seeded in 6 replicate wells per group, and each experiment was independently repeated three times. Briefly, the initial culture medium was removed, and a mixture of CCK‐8 reagent and fresh medium (in a ratio of 1:10) was added to each well. Following this, the cells were incubated for 2 h. Absorbance was subsequently measured at a wavelength of 450 nm using a microplate reader.

### 2.2 In Vivo Study of TNF‐α Inducing Pyroptosis in Diabetic Rats

#### 2.2.1 Animals and Experimental Design

**FIGURE 1:**
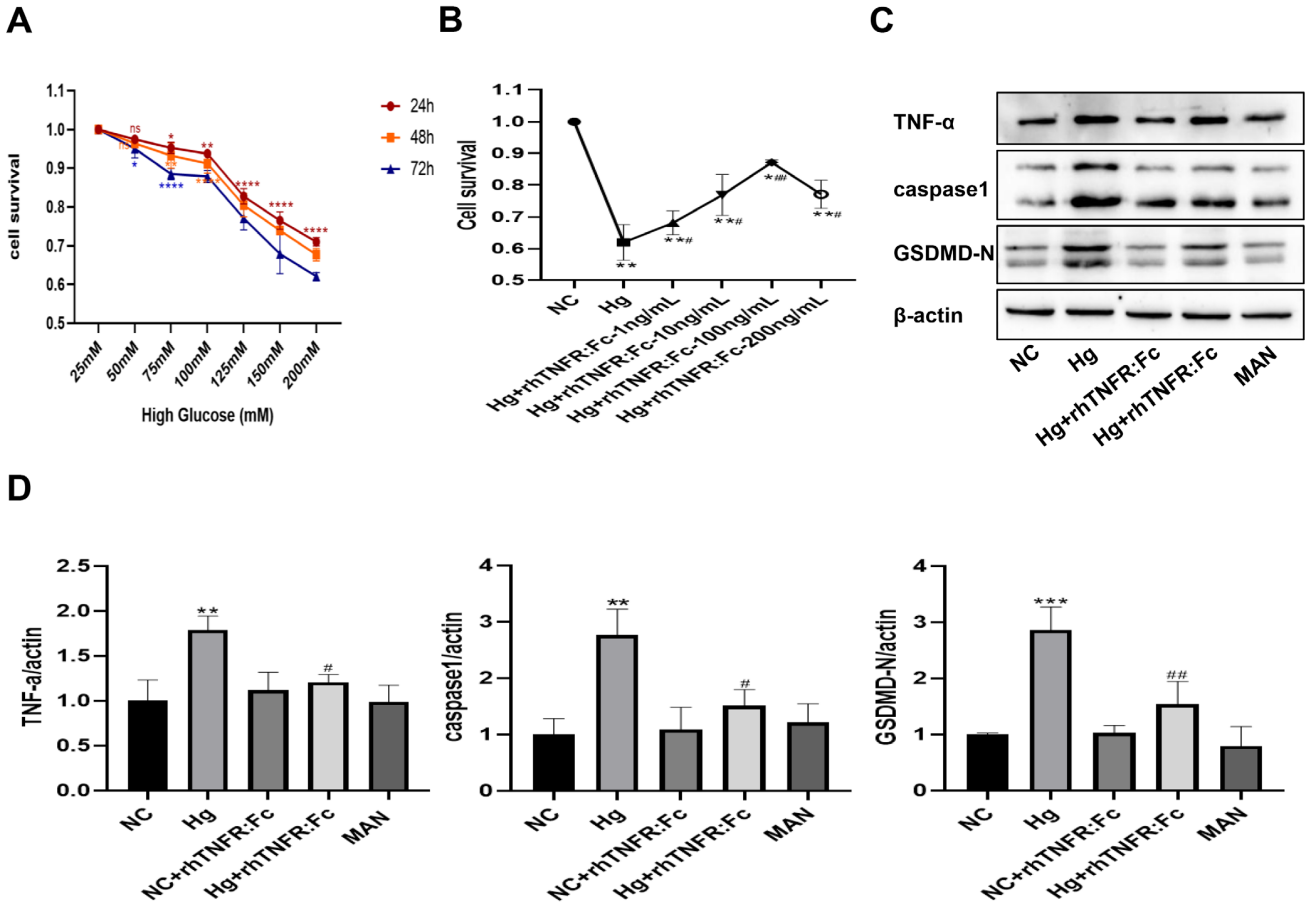
Inhibition of TNF‐α protects HepG2 cells from high glucose‐induced pyroptosis. (A) CCK‐8 assay verifying the impact of high glucose on HepG2 cell viability. (B) CCK‐8 assay verifying the effects of different TNF‐α inhibitors on high‐glucose‐treated HepG2 cells. (C) Western blotting image showing TNF‐α, caspase1, and GSDMD‐N protein expression. (D) TNF‐α, caspase1 and GSDMD‐N protein quantitative analysis. Data are expressed as mean ± standard deviation (SD), with *n* = 3 per group. Compared with the NC group and 25 mM: **p < 0.01, ***p < 0.001，****p < 0.0001. Compared with the Hg group: ^#^ *p* < 0.05, ^##^ *p* < 0.01. NC, normal control; HG, high glucose; MAN, mannitol; rhTNFR:Fc (TNF‐α inhibitor) All protein expression levels have been normalized to the NC group.

Male Sprague–Dawley rats (6–7 weeks old, 200–230 g) were obtained from the Experimental Animal Centre of the Second Affiliated Hospital of Harbin Medical University and maintained under ventilated conditions at approximately 23°C with a 12 h light/dark cycle, with free access to water and standard chow at the Animal Experiment Centre of Jiamusi University. All procedures were approved by the Experimental Animal Management and Ethics Committee of Jiamusi University (Approval No. JDJCYXY2022001) and conducted in accordance with relevant guidelines. After 1 week of acclimatization, T1DM was induced by a single intraperitoneal injection of streptozotocin (STZ; 60 mg/kg in citrate buffer, pH 4.2–4.5). Rats were fasted for 12 h before STZ administration. Fasting blood glucose (FBG) was measured 72 h post‐injection, and diabetes was confirmed if FBG ≥ 16.7 mM. As outlined in Figure 1, rhTNFR:Fc treatment began 1 day before STZ injection and continued twice weekly for 10 weeks. Rats were divided into four groups (*n* = 8/group): Con: received citrate buffer only; Dia: diabetic; Dia + rhTNFR:Fc: diabetic, treated with rhTNFR:Fc (2 mg/kg, subcutaneously, twice weekly); Con + rhTNFR:Fc: non‐diabetic, received the same rhTNFR:Fc regimen. After 10 weeks, rats were anaesthetised with isoflurane. Blood was collected via cardiac puncture, and euthanasia was performed by decapitation. Liver tissues were harvested, rinsed in PBS, and either snap‐frozen for molecular analysis or fixed in 4% paraformaldehyde for histology.

#### 2.2.2 Measurement of Blood Glucose and Body Weight

Prior to euthanasia, fasting blood glucose levels were measured in each rat using a blood glucose meter (Safe AQ proIII, Sannuo, China). The body weight and liver weight of each rat were also recorded.

#### 2.2.3 Measurement of Biochemical Parameters

Blood samples were collected prior to euthanasia and subsequently centrifuged at 3000 g for 5 min in a low‐temperature centrifuge (Model TDL‐50B, Anting, Shanghai). The supernatant was collected, and the levels of alkaline phosphatase (ALP), triglycerides (TG), aspartate aminotransferase (AST), and alanine aminotransferase (ALT) were measured using an automated biochemical analyser (BS‐480, Mindray, China).

#### 2.2.4 Histopathological Examination

Fixed liver tissues were rinsed overnight under running water, followed by paraffin embedding and sectioning into 3‐μm thick slices. The sections were dewaxed, rehydrated, and stained with haematoxylin and eosin (H&E; C0105S, Beyotime, China). Subsequently, the slices were dehydrated through a graded ethanol series, cleared in xylene, and mounted. Histopathological changes were ultimately examined under an optical microscope (DM4000B, Leica, Germany).

#### 2.2.5 Periodic Acid‐Schiff (PAS) Staining

PAS staining was performed using a commercial kit (C0142S, Beyotime, China) according to the manufacturer's instructions. Briefly, liver tissue sections were oxidized with periodic acid solution for 10 min at room temperature in a light‐protected humidified chamber, followed by incubation with Schiff's reagent for 1 h at 37°C in the dark. The sections were then counterstained with haematoxylin for 30 s, differentiated, and mounted for microscopic examination.

#### 2.2.6 Western Bloting Analysis

Total protein was extracted from liver tissues and HepG2 cells using RIPA lysis buffer (Beyotime, P0013B) containing PMSF (Beyotime, ST505) and phosphatase inhibitors. For liver tissues, homogenates were incubated at 4°C for 1 h and then centrifuged at 15,000 × g for 15 min at 4°C. For cells, lysis was performed on ice. Protein concentrations in the supernatants were determined using a BCA assay kit (Beyotime, P0010). Equal amounts of protein were separated by SDS‐PAGE and transferred onto PVDF membranes (Beyotime, P0021). After blocking with 5% skim milk in TBST for 1 h at room temperature, the membranes were incubated overnight at 4°C with the following primary antibodies: TNF‐α (1:400, Abmart, PY19810), HMGB1 (1:1500, Abmart, T55060), TLR4 (1:1300 for cells, Abmart, T61519; 1:1000 for tissues, Santa Cruz, sc‐52,962), MyD88 (1:1200, Abmart, PA1779), p65 (1:2500, Abmart, T55034), p‐p65 (1:2000, Abmart, TP56372), caspase1 (1:2500, Abcam, ab179515), GSDMD‐N (1:3300, Abmart, PU224937), ASC (1:900, WanLei, WL02462, tissues only), NLRP3 (1:800, Abmart, TB4673, tissues only), IL‐1*β* (1:1500, Abcam, ab283818, tissues only), IL‐18 (1:1100, Abmart, M027287, tissues only), and GSDMD (1:1000, Santa Cruz, sc‐376,318, tissues only). After washing with TBST, membranes were incubated with HRP‐conjugated secondary antibodies (Boster, BA1039, 1:7500; BA1058, 1:7000) for 1 h at room temperature. Protein bands were visualized using an ECL substrate (Beyotime, P0018) and imaged with a Tanon‐5200 system. Band intensities were quantified using ImageJ software (v1.8.0).

#### 2.2.7 Analytical Methods

The analysis and visualization of data were conducted utilizing GraphPad Prism version 8.0 (GraphPad Software, San Diego, CA, United States). Results are presented as mean ± standard deviation (SD). Data obtained from both in vivo and in vitro experiments were assessed through one‐way analysis of variance (ANOVA), followed by Tukey's test for assessing multiple comparisons. *p* < 0.05 was considered statistically significant.

## 3 Results

### 3.1 In Vitro Experiments

#### 3.1.1 Inhibition of TNF‐α Protects HepG2 Cells From High Glucose‐Induced Pyroptosis

To evaluate the role of TNF‐α in high glucose‐induced pyroptosis, a cellular injury model was first established. Treatment of HepG2 cells with high glucose resulted in a time‐ and concentration‐dependent decrease in cell viability. A concentration of 75 mM for 48 h was selected to establish the model, as this condition induced significant cytotoxicity while retaining a sufficient number of cells for subsequent experiments (Figure 1A). To elucidate the involvement of TNF‐α, cells were pretreated with different concentrations of the TNF‐α inhibitor rhTNFR:Fc prior to high glucose stimulation. This pretreatment alleviated high glucose‐induced cell injury in a concentration‐dependent manner, with the highest cell survival rate observed at 100 ng/mL; consequently, this optimal concentration was selected for subsequent mechanistic studies(Figure 1B). The results demonstrated that high glucose stimulation significantly increased the protein expression levels of GSDMD‐N, caspase1, and TNF‐α, whereas inhibition of TNF‐α markedly reversed the upregulation of these proteins. These results collectively confirm the critical role of TNF‐α in driving high glucose‐induced pyroptosis. Finally, the use of a mannitol control (MAN) verified that the observed effect was specific to the high glucose environment and not a general response to hyperosmotic stress (Figure 1C,D).

#### 3.1.2 TNF‐α Induces Pyroptosis by Activating the HMGB1‐Mediated TLR4/MyD88/NF‐κB Pathway

**FIGURE 2:**
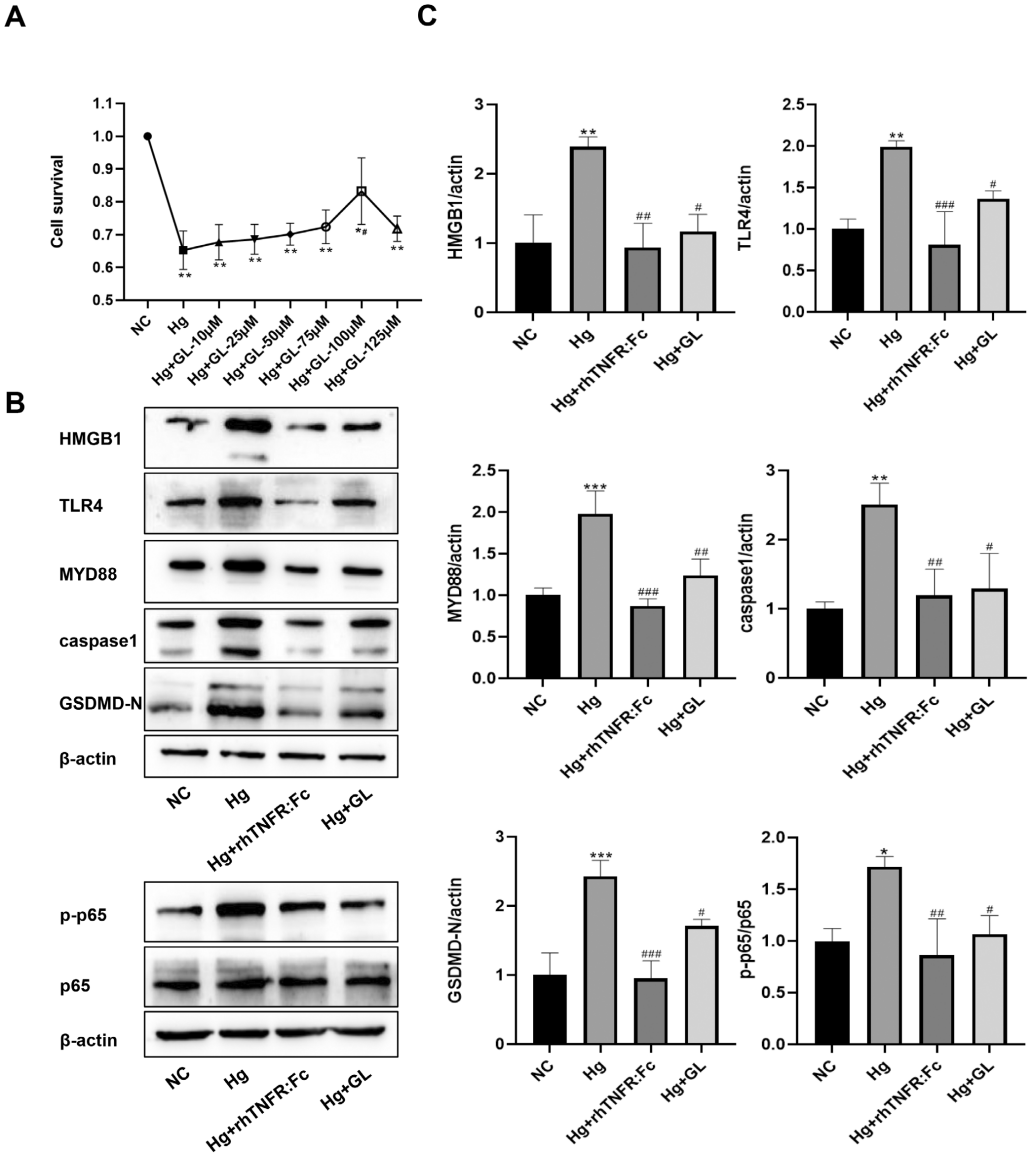
TNF‐α induces pyroptosis by activating the HMGB1‐mediated TLR4/MyD88/NF‐κB pathway in HepG2 cells. (A) CCK‐8 assay verifying the cell viability of different HMGB1 inhibitors on HepG2 cells after high glucose treatment. (B) Western blotting images of HMGB1, TLR4, MyD88, p‐p65, p65, caspase 1, and GSDMD‐N protein expression. (C) Quantitative analysis of HMGB1, TLR4, MyD88, p‐p65/p65, caspase1 and GSDMD‐N protein expression. Data are expressed as mean ± (SD), with *n* = 3 per group. Compared with the NC group: **p < 0.01, ***p < 0.001, ****p < 0.0001; Compared with the Hg group: ^#^ *p* < 0.05, ^##^ *p* < 0.01. NC, normal control; HG, high glucose; GL, glycyrrhetinic acid (HMGB1 inhibitor); rhTNFR:Fc (TNF‐α inhibitor). All protein expression levels have been normalized to the NC group.

Based on the finding that inhibition of TNF‐α downregulates HMGB1 and its downstream pathway, we further investigated whether HMGB1 is an essential mediator in this process. Our data indicate that under high glucose conditions, TNF‐α is activated and subsequently triggers a pro‐pyroptotic signalling cascade. To determine whether HMGB1 acts as a critical downstream mediator of TNF‐α in this cascade, GL was used to directly inhibit HMGB1. The results showed that GL pretreatment increased the viability of HG‐induced HepG2 cells in a concentration‐dependent manner, with the highest cell survival rate observed at 100 μM(Figure 2A); this optimal concentration was therefore used in all subsequent mechanistic experiments. Compared with the untreated HG group, HMGB1 inhibition in the HG + GL group significantly suppressed the HG‐induced upregulation of TLR4, MyD88, and the p‐p65/p65 ratio. Correspondingly, the enhanced expression of key pyroptosis execution proteins, GSDMD‐N and caspase1, was also markedly reduced following HMGB1 inhibition (Figure 2B,C). Collectively, these findings demonstrate that high glucose‐activated TNF‐α promotes the TLR4/MyD88/NF‐κB signalling pathway and subsequent pyroptosis in an HMGB1‐dependent manner. Pharmacological inhibition of HMGB1 efficiently interrupts this signalling cascade, supporting that HMGB1 functions as an indispensable downstream mediator of TNF‐α in high glucose‐induced hepatic cell injury.

### 3.2 In Vivo Experiments

#### 3.2.1 Inhibition of TNF‐α Ameliorates Metabolic Parameters Without Altering Hyperglycemia in a Type 1 Diabetic Rat Model

**FIGURE 3:**
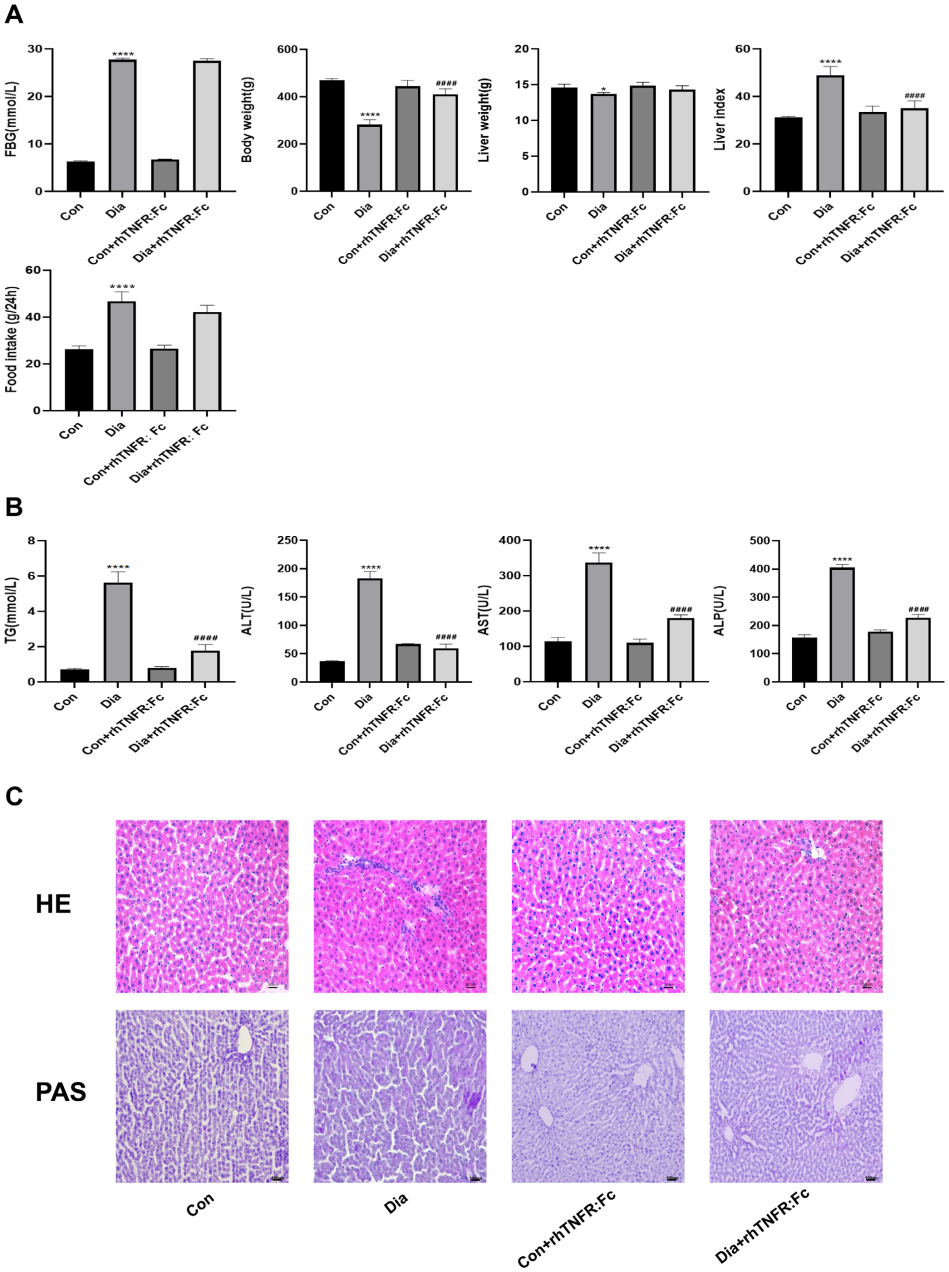
Inhibition of TNF‐α ameliorates liver injury and exerts a protective effect on the liver. (A) Fasting blood glucose, Body weight, Liver weight, Liver index and Food intake parameters. (B) TG content, ALT content, AST content and ALP content parameters. (C) HE staining of liver tissue. Magnification: 200× and PAS staining of liver tissue. Magnification: 100×. Data are expressed as mean ± (SD), *n* = 5 per group. Compared with the Con group: *p < 0.05, ****p < 0.0001. Compared with the Dia group: ^####^ *p* < 0.0001. Con, control group; Dia, diabetic group; rhTNFR:Fc, TNF‐α inhibitor treatment group.

As shown in Figure 3A, the Dia group exhibited significantly higher FBG and liver index levels compared with the Con group, while body weight and liver weight were significantly lower, indicating successful model establishment with evident metabolic disorder. To exclude potential confounding effects of appetite and nutritional intake, 24 h food intake was continuously monitored throughout the entire 10 week diabetic modelling period, and average daily food intake was compared among all groups. No significant differences in food intake were observed between the Con and Con+rhTNFR:Fc groups, or between the Dia and Dia + rhTNFR:Fc groups. Therefore, the body weight and related phenotypic changes observed in this study are independent of dietary intake and nutritional status and cannot be explained by differences in appetite or food consumption. Following rhTNFR:Fc intervention, body weight in the Dia + rhTNFR:Fc group was significantly increased compared with the Dia group, and the liver index was also significantly reduced, suggesting that TNF‐α inhibition partially reverses diabetes‐induced weight loss and hepatic metabolic abnormalities. However, no statistically significant differences in liver weight or FBG levels were observed between the Dia + rhTNFR:Fc and Dia groups, indicating that the protective effects of rhTNFR:Fc may not depend on glucose‐lowering or direct liver weight gain. Furthermore, no notable differences in body weight, liver weight, FBG levels, or liver index were observed between the Con+rhTNFR:Fc and Con groups, confirming that rhTNFR:Fc does not induce significant metabolic changes under non‐diabetic conditions.

#### 3.2.2 TNF‐α Inhibition Improves Biochemical Indicators of Liver Injury

To evaluate the protective effect of TNF‐a inhibition on the liver in diabetic rats, we assessed serum markers of liver injury. Biochemical analysis revealed that the levels of TG, ALT, AST, and ALP were significantly elevated in the Dia group compared to the Con group. Treatment with rhTNFR:Fc significantly reduced the levels of these markers in the Dia + rhTNFR:Fc group compared to the untreated Dia group. No significant differences in these parameters were observed between the Con and Con+rhTNFR:Fc groups (Figure 3B).

#### 3.2.3 TNF‐α Antagonism Alleviates Hepatic Histopathological Damage and Reduces Glycogen Accumulation in the Liver of Diabetic Rats

To assess the effect of TNF‐α inhibition on pathological changes in the liver, we examined liver tissue morphology using H&E staining. Light microscopy revealed a normal liver tissue structure in the Con group rats. Hepatocytes were arranged in an orderly manner with uniform cytoplasmic staining, and nuclei were distinct and deeply basophilic. In contrast, the Dia group exhibited severe histopathological abnormalities, including extensive inflammatory cell infiltration, hepatocyte edema, and dilated hepatic sinusoids. Treatment with rhTNFR:Fc significantly reduced inflammatory infiltration and improved hepatocyte edema and architecture compared to the Dia group. The Con+rhTNFR:Fc group showed no significant pathological alterations and was comparable to the Con group. These results indicate that blocking TNF‐α ameliorates diabetes‐induced hepatic histopathological damage. Additionally, PAS staining revealed a significant increase in PAS‐positive material in the Dia group compared to the Con group. This accumulation was markedly reduced by rhTNFR:Fc treatment in the Dia + rhTNFR:Fc group (Figure 3C).

#### 3.2.4 Suppression of TNF‐α Attenuates NLRP3 Inflammasome‐Dependent Hepatocyte Pyroptosis in Diabetic Rats

**FIGURE 4:**
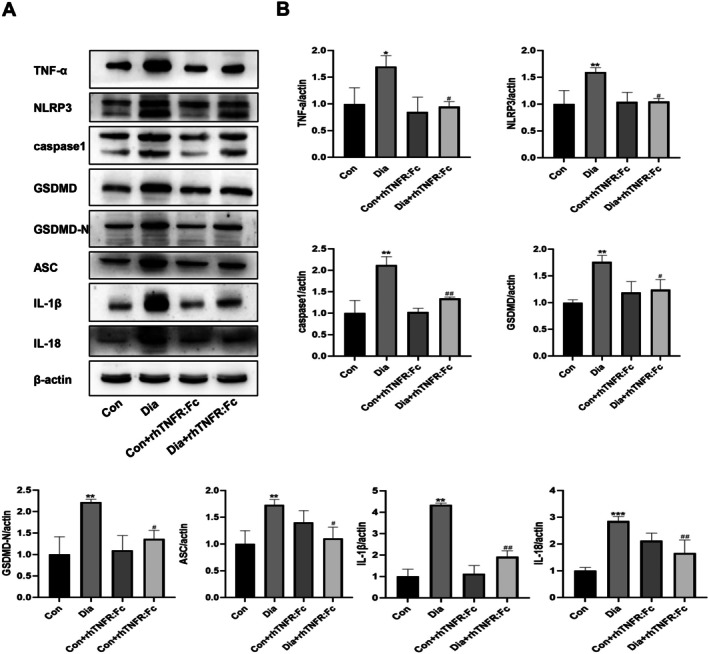
Inhibition of TNF‐α on pyroptosis in type 1 diabetic rats. (A) Western blotting images showing TNF‐α, NLRP3, caspase1, GSDMD, GSDMD‐N, ASC, IL‐1*β*, and IL‐18 protein expression. (B) Quantitative analysis of TNF‐α, NLRP3, caspase1, GSDMD, GSDMD‐N, ASC, IL‐1β, and IL‐18 protein levels. Data are expressed as mean ± (SD), *n* = 3 per group. Compared with the Con group: *p < 0.05, **p < 0.01, ***p < 0.001, ****p < 0.0001. Compared with the Dia group: ^#^ *p* < 0.05, ^##^ *p* < 0.01, ^####^ *p* < 0.0001. All protein expression levels have been normalized to the Con group.

Analysis of TNF‐α protein levels in liver tissue showed a significant increase in the Dia group compared to the Con group. This elevated level was significantly reduced in the Dia + rhTNFR:Fc group. No statistically significant difference was found between the Con and Con+rhTNFR:Fc groups. We next investigated the expression of proteins related to pyroptosis. Compared with the Con group, the Dia group exhibited elevated protein expression of the NLRP3 inflammasome components NLRP3 and ASC. Additionally, the expression of pyroptosis‐associated proteins, including GSDMD/GSDMD‐N, caspase1, IL‐1*β*, and IL‐18, was significantly elevated. Following rhTNFR:Fc treatment, the expression of these inflammasome and pyroptosis‐related proteins was markedly decreased in the Dia + rhTNFR:Fc group compared to the Dia group. In contrast, no significant differences in the expression of these proteins were observed between the Con and Con+rhTNFR:Fc groups. These results indicate that diabetes is associated with the activation of the NLRP3 inflammasome and pyroptosis in the rat liver, and that inhibition of TNF‐α by rhTNFR:Fc reduces the expression of key proteins in this pathway (Figure 4A,B).

#### 3.2.5 TNF‐α Blockade Downregulates HMGB1 and Suppresses the HMGB1‐Mediated TLR4/MyD88/NF‐κB Signalling Pathway

**FIGURE 5:**
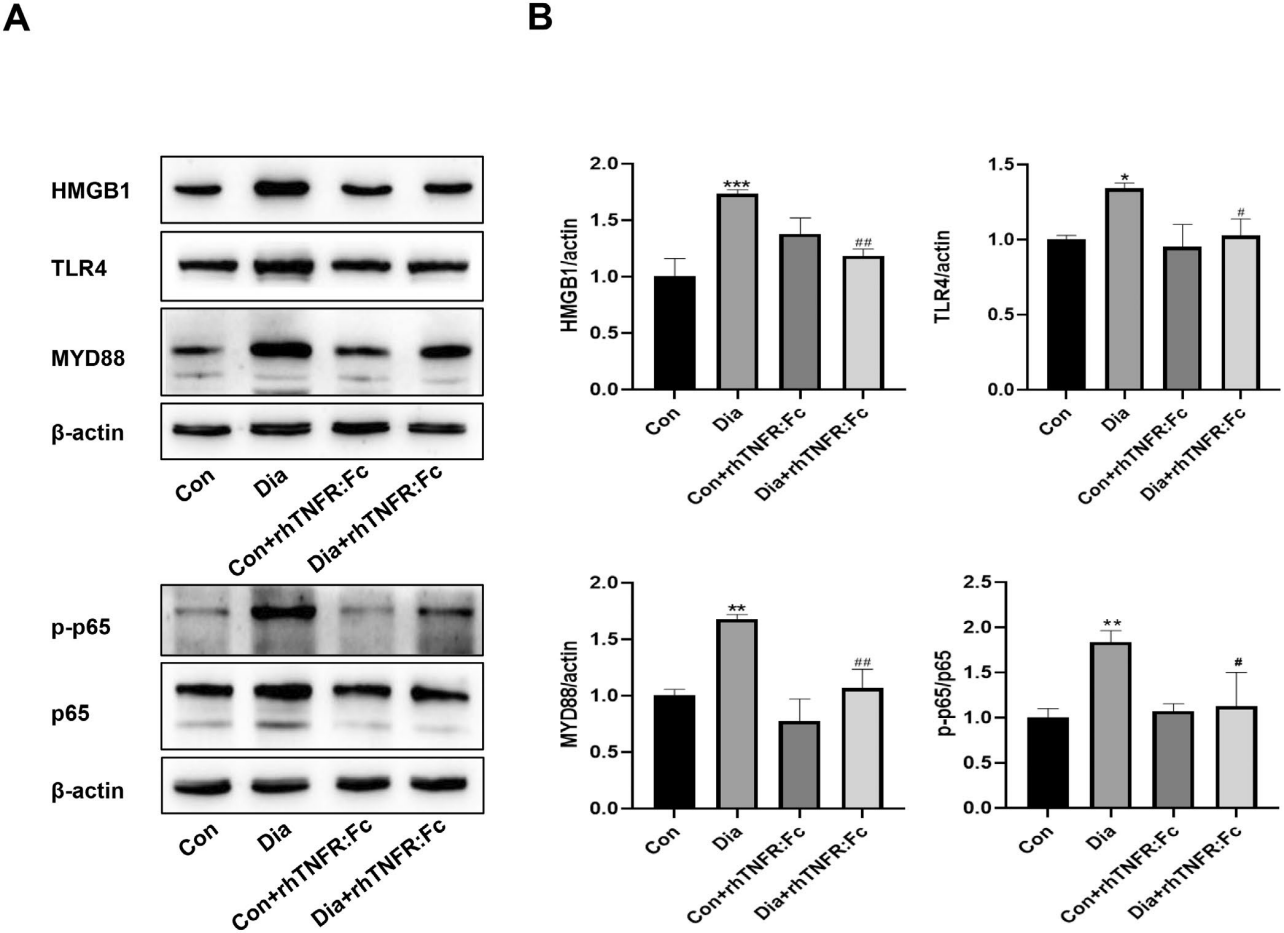
Inhibition of TNF‐α on HMGB1 and TLR4/MyD88/NF‐κB signalling pathway in type 1 diabetic rats. (A) Western blotting images showing HMGB1, TLR4, MyD88, pp65, and p65 protein expression. (B) Quantitative analysis of HMGB1, TLR4, MyD88, and p‐65/p65 protein levels. Data are expressed as mean ± (SD), *n* = 3 per group. Compared with the Con group: *p < 0.05, **p < 0.01, ***p < 0.001. Compared with the Dia group: ^#^ *p* < 0.05, ^##^ *p* < 0.01. All protein expression levels have been normalized to the Con group.

HMGB1 protein levels were significantly higher in the Dia group than in the Con group. This increase was significantly attenuated by rhTNFR:Fc treatment in the Dia + rhTNFR:Fc group. In contrast, HMGB1 expression did not differ significantly between the Con+rhTNFR:Fc and Con groups. Similarly, the protein levels of key components in the TLR4/MyD88/NF‐κB signalling pathway were significantly upregulated in the Dia group compared to the Con group. rhTNFR:Fc treatment also markedly downregulated the expression of these pathway proteins in the Dia + rhTNFR:Fc group compared to the untreated Dia group. No significant differences in the expression of these proteins were observed between the Con+rhTNFR:Fc and Con groups (Figure 5A,B). These results indicate that both HMGB1 and the TLR4/MyD88/NF‐κB signalling pathway are activated in the diabetic liver, and that inhibition of TNF‐α significantly suppresses their expression.

## 4 Discuss

DLI is a serious complication closely associated with chronic inflammation [19, 20]. As a central metabolic organ, the liver plays a critical role in glucose homeostasis, rendering it a primary target for diabetic complications [21]. Hyperglycemia significantly increases the risk of liver injury and fibrosis, which may progress to cirrhosis and hepatocellular carcinoma [22]. Therefore, establishing effective liver protection strategies is crucial for improving the prognosis of diabetic patients. TNF‐α, a key pro‐inflammatory cytokine, is upregulated during insulin resistance and diabetes and participates in various hepatic pathological processes [23]. Notably, previous studies have indicated that TNF‐α inhibition alleviates pyroptosis in other disease models. Based on this evidence, the present study aimed to investigate the protective effect of TNF‐α inhibition against DLI and its underlying mechanism.

Our initial in vitro study established a clear link between TNF‐α and pyroptosis, demonstrating that TNF‐α drives pyroptosis in HepG2 cells under high‐glucose conditions. Using a TNF‐α inhibitor, we found that blockade significantly reduced the upregulation of the pyroptosis executor proteins GSDMD‐N and caspase1, indicating a crucial role for TNF‐α in initiating inflammatory hepatocyte death under diabetic conditions. Importantly, hyperosmotic controls ruled out nonspecific stress effects, highlighting the specificity of the glucose–TNF‐α–pyroptosis axis.

Subsequent in vivo studies robustly validated these findings in a physiologically relevant context. We demonstrated that TNF‐α inhibition via rhTNFR:Fc conferred significant protection against DLI in a type 1 diabetic rat model. The treatment ameliorated body and liver weight loss, attenuated histopathological damage, and improved serum markers of liver injury (ALT, AST, ALP, TG) – all without altering blood glucose levels. This key finding indicates that the hepatoprotective effect of TNF‐α inhibition is independent of glycemic control. Furthermore, the absence of significant differences in food intake among groups excluded the possibility that the observed metabolic improvements were secondary to altered appetite or nutritional status. This further underscores its potential as a direct therapeutic strategy for DLI.

To further elucidate the cellular mechanism underlying this protection, we investigated pyroptosis in vivo. Pyroptosis, an inflammatory form of programmed cell death, contributes to liver injury via NLRP3 inflammasome activation [12, 24, 25]. Both the canonical and non‐canonical pyroptosis pathways converge on GSDMD cleavage, leading to plasma membrane pore formation and the release of IL‐1*β* and IL‐18 [26, 27]. Our results showed significant activation of pyroptosis in the livers of diabetic rats, as evidenced by upregulated expression of NLRP3, caspase1, GSDMD, and IL‐1*β*. TNF‐α inhibition effectively reversed this activation, establishing the pivotal role of pyroptosis in DLI and identifying TNF‐α as an upstream regulator of hepatocyte pyroptosis.

**FIGURE 6:**
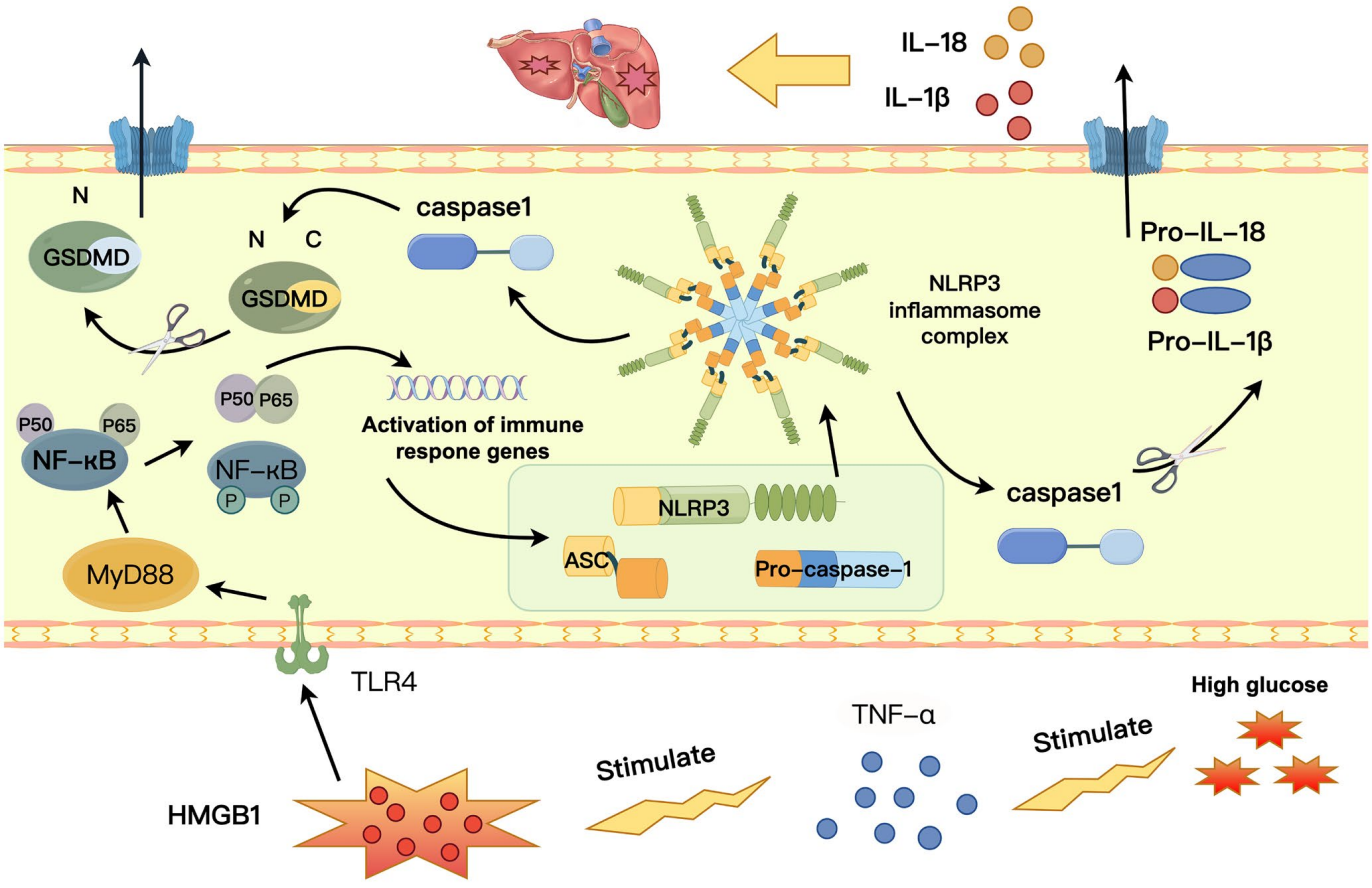
Schematic diagram of the proposed mechanism. TNF‐α promotes pyroptosis in diabetic liver injury via the HMGB1/TLR4/MyD88/NF‐κB pathway.

To systematically delineate the intermediate steps by which TNF‐α regulates pyroptosis, we focused on downstream signalling events. Previous studies have indicated that the TLR4/MyD88/NF‐κB pathway promotes liver disease progression and is closely associated with pyroptosis during hepatic ischemia–reperfusion injury [28, 29]. Our results demonstrated marked activation of this pathway under diabetic conditions, which was suppressed by rhTNFR:Fc, suggesting that TNF‐α acts upstream. Normally, HMGB1 is primarily nuclear and chromatin‐bound; however, under stress conditions, it translocates to the cytoplasm and extracellular space. As a damage‐associated molecular pattern (DAMP) molecule, HMGB1 mediates inflammatory and immune responses [30, 31]. Our data revealed that HMGB1 expression was significantly increased under high‐glucose conditions, and this increase was reversed by TNF‐α inhibition, identifying HMGB1 as a key downstream mediator of TNF‐α signalling. Crucially, experiments with an HMGB1 inhibitor confirmed that its blockade not only suppressed TLR4/MyD88/NF‐κB pathway activation but also significantly alleviated pyroptosis. Collectively, our data delineate a coherent signalling cascade in DLI: TNF‐α promotes HMGB1 release/activation, which subsequently drives the TLR4/MyD88/NF‐κB pathway, leading to NLRP3 inflammasome assembly and ultimately GSDMD‐mediated hepatocyte pyroptosis(Figure 6). This study systematically elucidates the central role of the TNF‐α–HMGB1/TLR4/MyD88/NF‐κB–pyroptosis axis in DLI, providing a novel theoretical framework for its pathology and highlighting potential therapeutic targets. However, this study has limitations. Although we focused on the TLR4 pathway, the potential involvement of other HMGB1 receptors (e.g., RAGE) requires further exploration. The specific upstream mechanisms by which TNF‐α regulates HMGB1 release remain incompletely understood, and the clinical translational potential of rhTNFR:Fc warrants validation in larger‐scale trials. Future research should validate the generalizability of this pathway in additional models and develop specific inhibitors targeting its key nodes to inform combination therapies for DLI.

## Author Contributions

Conceptualization, Dayin Chen and Jiajun Cheng; methodology, Bin Ni; software, Ruixin Zhu; validation, Jiajun Cheng, Yihe Cao and Ruixin Zhu; formal analysis, Wei Wu; investigation, Minghui Zhang; resources, Bin Ni; data curation, Dayin Chen; writing – original draft preparation, Jiajun Cheng; writing – review and editing, Hongyu Sui; visualization, Wei Wu; supervision, Zhimei Jiang; project administration, Hongyu Sui; funding acquisition, Dayin Chen. All authors have read and agreed to the published version of the manuscript.

## Funding

This research was funded by the Natural Science Foundation of Heilongjiang Province (grant no. PL2024C006), Northern Unique Medicinal Resources Research and Development Team (grant no. DJXSTD202403), Jiamusi University Doctoral Cultivation Fund (JMSUBZ2021‐13) and Training Project for Innovation and Entrepreneurship of Jiamusi University (grant no. 202410222027).

## Conflicts of Interest

The authors declare no conflicts of interest.

## Data Availability

The data that support the findings of this study are available from the corresponding author upon reasonable request.
